# The dynamics of sperm cooperation in a competitive environment

**DOI:** 10.1098/rspb.2014.0296

**Published:** 2014-09-07

**Authors:** Heidi S. Fisher, Luca Giomi, Hopi E. Hoekstra, L. Mahadevan

**Affiliations:** 1 Department of Organismic and Evolutionary Biology, Harvard University, Cambridge, MA 02138, USA; 2 Howard Hughes Medical Institute, Department of Molecular and Cellular Biology, Museum of Comparative Zoology, Harvard University, Cambridge, MA 02138, USA; 3 School of Engineering and Applied Sciences, Harvard University, Cambridge, MA 02138, USA; 4 Department of Physics, Kavli Institute for Nanobio Science and Technology, Wyss Institute for Bioinspired Engineering, Harvard University, Cambridge, MA 02138, USA; 5 SISSA, International School for Advanced Studies, Trieste, Italy

**Keywords:** sperm aggregation, mathematical model, sperm motility, sperm competition, sexual selection, collective motion

## Abstract

Sperm cooperation has evolved in a variety of taxa and is often considered a response to sperm competition, yet the benefit of this form of collective movement remains unclear. Here, we use fine-scale imaging and a minimal mathematical model to study sperm aggregation in the rodent genus *Peromyscus*. We demonstrate that as the number of sperm cells in an aggregate increase, the group moves with more persistent linearity but without increasing speed. This benefit, however, is offset in larger aggregates as the geometry of the group forces sperm to swim against one another. The result is a non-monotonic relationship between aggregate size and average velocity with both a theoretically predicted and empirically observed optimum of six to seven sperm per aggregate. To understand the role of sexual selection in driving these sperm group dynamics, we compared two sister-species with divergent mating systems. We find that sperm of *Peromyscus maniculatus* (highly promiscuous), which have evolved under intense competition, form optimal-sized aggregates more often than sperm of *Peromyscus polionotus* (strictly monogamous), which lack competition. Our combined mathematical and experimental study of coordinated sperm movement reveals the importance of geometry, motion and group size on sperm velocity and suggests how these physical variables interact with evolutionary selective pressures to regulate cooperation in competitive environments.

## 1. Introduction

The factors that contribute to reproductive success are numerous and complex, yet across vertebrates, relative sperm motility is often the best predictor of male fertility [1–7]. When competition among males intensifies, adaptations that improve sperm swimming performance are therefore expected to be strongly favoured [8,9]. Indeed, comparisons between related taxa reveal that sperm of polyandrous species, in which females mate with multiple partners during a reproductive cycle, swim faster than sperm from closely related monogamous species [10,11]. Among the many strategies that improve sperm swimming performance, perhaps the most intriguing mechanism involves cooperation or association with other motile cells [12]. Even without direct attachment, sperm of some species interact with one another via flow fields that result from hydrodynamic interactions [13]. These associations, however, are magnified when multicellular groups form by conjugation, ranging in size from sperm pairs to large aggregates containing hundreds of sperm (reviewed in [14,15]). Sperm aggregation is often assumed to improve motility, yet comparative studies have shown inconsistent results (reviewed in [14,15]), and the underlying mechanics of the associations remain largely unknown.

**Figure 1.**
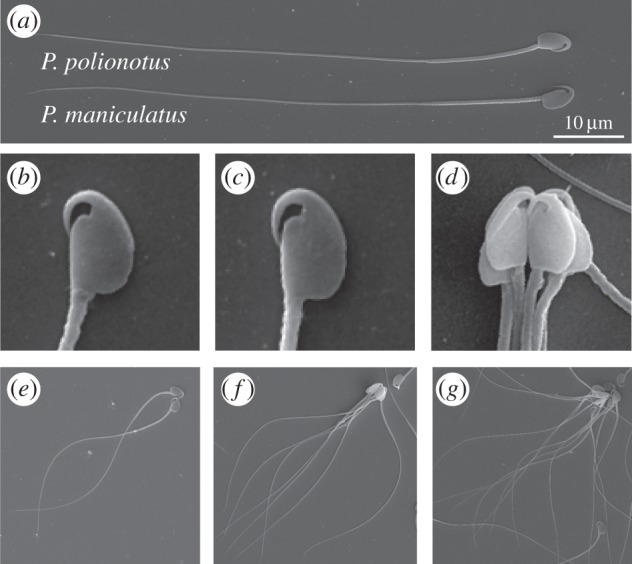
Scanning electron micrographs of (*a*) whole *Peromyscus* sperm cells, and head morphology of a single (*b*) *P. maniculatus* and (*c*) *P. polionotus* sperm. (*d*) Head orientation of sperm in a typical aggregate with hooks facing inward, and aggregates consisting of (*e*) two, (*f*) seven and (*g*) thirteen *P. maniculatus* cells.

**Figure 2.**
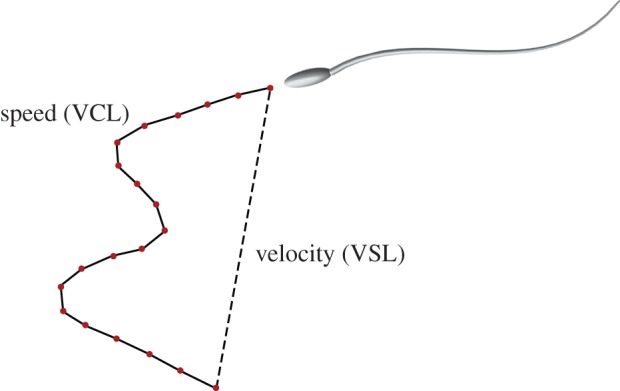
Schematic of the average velocity (VSL) and speed (VCL). VSL is calculated by dividing the distance between the initial and final position in a sperm trajectory (dashed line) by the time Δ*t* employed to move; VCL is found by dividing the length of the actual curvilinear trajectory (solid line) by Δ*t*. (Online version in colour.)

Like most muroid rodents, sperm from mice in the genus *Peromyscus* typically possess an apical hook on the head (figure 1*a–c*) [16] that is thought to facilitate the formation [17] and/or stabilization [18] of sperm groups (but see [19]). Aggregations of *Peromyscus* sperm cells are formed by secondary conjugation [12]: sperm are ejaculated as solitary cells, but quickly begin to form multicellular aggregates by adhering to one another at or near the hook (figure 1*d*) [20]. Overall, these motile sperm groups have a larger average velocity (straightline velocity, VSL; figure 2) than single cells; however, the largest groups, those over twenty cells, are often not motile at all [20]. Understanding how sperm aggregates achieve greater average velocity than single cells, whether by increasing their speed (curvilinear velocity, VCL; figure 2) or travelling in a straighter trajectory (linearity), and how group size can hinder motility, is key to understanding how post-copulatory male–male competition may be acting on sperm behaviour to drive and constrain group formation.

In the genus *Peromyscus*, sperm competition is predicted to be greatest in *P. maniculatus,* because both sexes mate with multiple partners, often in overlapping series just minutes apart [21], and females frequently carry multiple-paternity litters in the wild [22]. By contrast, its sister species, *Peromyscus polionotus,* is strictly monogamous on the basis of both behavioural [23] and genetic data [24]. The sperm of both species form aggregations with similar geometry and cell orientation, probably owing to analogous morphology of their sperm heads [25], yet the competitive environments experienced by *P. maniculatus* and *P. polionotus* sperm represent divergent selective regimes, which is believed to shape how cooperative sperm groups assemble [20]. Here, we use a minimal mathematical model to predict how sperm can improve their average velocity by forming aggregations and then use fine-scale imaging to test these predictions and gain a deeper understanding of how sexual selection has acted on this unique form of cooperation in *Peromyscus* sperm.

## 2. Material and methods

### (a) Mathematical model

A simple mechanistic picture of how the average velocity of sperm is a non-monotonic function of aggregate size is suggested by the geometry of the aggregates shown in figure 1*e–g*. As sperm cells form small oriented clusters, their motive force and cluster geometry can increase owing to the collective beating of their flagella that leads to a greater dynamical persistence. However, in large clusters, the geometry of the aggregate approaches that of an isotropic cluster so that their collective ability to move is severely hindered. A minimal model described below allows us to quantify the advantage of cooperation in a competitive environment using observable physical variables.

Our approach follows a set of models originally developed for flocking behaviour of organisms [26,27], which have been used successfully to describe collective motion in a variety of natural and artificial systems, including fish and birds [28], insects [29], bacterial colonies [30] and robots [31] (for details, see appendix). In this spirit, we treat sperm as individual self-propelled particles [32] that can interact with each other geometrically and mechanically, consistent with the biology of *Peromyscus* sperm aggregation [20]. We restrict our attention to the dynamics of the aggregates once they form, not attempting to address the process of hydrodynamic self-organization itself. Our method relies on three basic assumptions: (i) although the flagellum is responsible for propulsion, it does not contribute to mechanical interactions between sperm; (ii) the main physical mechanism associated with aggregate formation is due to adhesion between sperm heads, consistent with our understanding of sperm morphology [12,20]; and (iii) hydrodynamic interactions between sperm in the aggregate are negligible. Thus, although hydrodynamic interactions among neighbouring sperm are important in creating self-organized patterns of swimming [33–35], in our minimal model that focuses on the dynamics of the aggregate, these interactions do not play a critical role.

**Figure 3.**
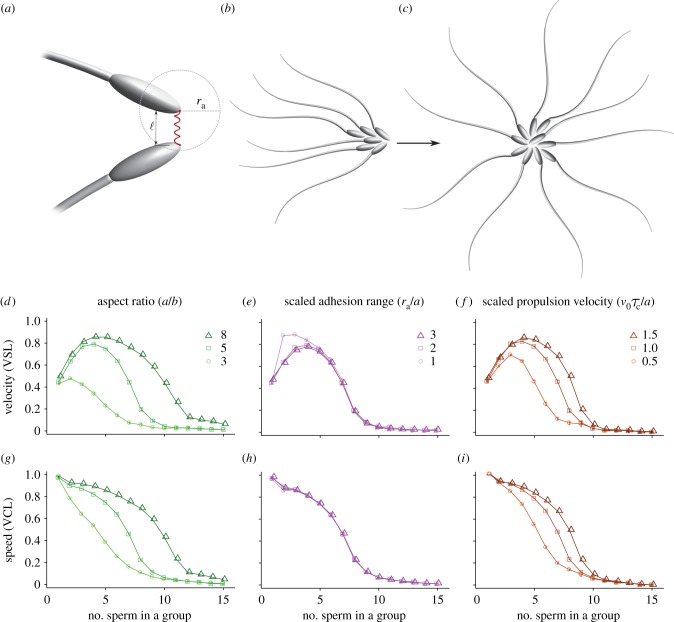
(*a*) Schematic of the adhesive interactions modelled in equation (2.2). Sperm heads are treated as self-propelled elliptical particles whose major and minor semiaxes have length *a* and *b*, respectively. Each particle is equipped with a given numbers of keys and locks, representing the adhesion complexes where the sperm can bind. When the key of a particle is within a certain distance *r*_a_ from the lock of another particle, a link represented by a linear spring is formed. The geometry of the aggregates affects their motility, so asymmetric aggregates (*b*) move fast and maintain a straight trajectory, whereas star-shaped aggregates (*c*) move slowly, because the velocities of the individual cells in the aggregate cancel each other. Average velocity (*d*–*f*) and speed (*g*–*i*) versus aggregate size obtained from a numerical integration of equations (2.1)–(2.5) for various aspect ratios *a*/*b* (*d*,*g*), scaled adhesion range *r*_a_/*a* (*e*,*h*), expressed in units of the particle major semiaxis length *a*, and the scaled propulsion velocity *v*_0_*τ_c_/a* (with *τ*_*c*_ = 1/*D* the timescale associated with the rotational noise) (*f*,*i*). (Online version in colour.)

With the aim of characterizing the empirical system using a small number of experimentally measurable parameters, we consider exclusively those features of sperm mechanics that are essential for the formation of motile aggregates. Thus, we note that individual sperm occupy space, are able to move and can link to other sperm. Single sperm cells are then represented as two-dimensional tailless elliptical particles that self-propel at constant velocity *v*_0_ in a plane in the direction of their major axis *n* while being subjected to random planar rotations. Each particle is assumed to have a given number of ‘keys’ and ‘locks’, representing the adhesion complexes on the sperm head. When the key of a particle is within a certain distance *r*_a_ from the lock of another particles, a link, represented by a linear spring of stiffness *k*_a_, is formed (figure 3*a*). If the key–lock distance eventually becomes larger than *r*_a_, the link breaks and the two sperm unbind (i.e. an individual adhesion complex can withstand forces up to a stall force *F*_a_ = *k*_a_*r*_a_). Finally, the particles are themselves assumed to be hard and unable to overlap, so that when in contact they pack as dictated by their geometry.

The above-described behaviour leads to equations of motion for the position of the *i*th sperm given by *r_i_*(*t*) and its orientation *θ*_*i*_(*t*) relative to the *x*-axis of the laboratory frame given by2.1
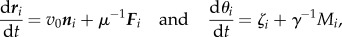
where the *i*th sperm has its major axis along ***n****_i_* = (cos *θ*_*i*_, sin *θ*_*i*_), ***F****_i_* is the total force acting on the *i*th particle resulting from the short-range steric interactions with the neighbours and adhesion:2.2
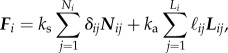
and *M_i_* is total torque acting on the *i*th particle:2.3
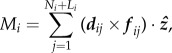
where *N_i_* is the number of neighbours of the *i*th particle, *L_i_* is the number of adhesive links, *k*_s_ is the elastic constant associated with steric interactions, *k*_a_ is the adhesive spring elastic constant, with 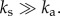 Furthermore, *δ*_*ij*_ and 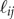 represent the length of the springs associated with the steric and adhesive interactions, with ***N****_ij_* and ***L****_ij_* unit vectors in the direction of the springs, *μ* and *γ* are translational and rotational drag coefficients, ***f****_ij_* is any of the force between the *i*th and *j*th cell appearing in equation (2.2) and ***d****_ij_* the associated lever arm, 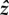 is the normal to the two-dimensional plane of motion and *ζ*_*i*_ is a zero-mean delta-correlated Gaussian random variable:2.4
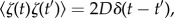
where *D* is a rotational diffusion coefficient. Here, we assume that the motion of the sperm is inertialess, consistent with the low Reynolds number regime they operate in, and further have ignored the effect of randomness in the translational degrees of freedom for the sake of simplicity.

Our minimal mechanistic model of interacting sperm captures the geometry of the individual sperm, their autonomous movement and finally their ability to interact with each other adhesively without overlap. While there are many possible variants of these models, the critical parameters in all of them will be qualitatively similar: the aspect ratio of the sperm head, the scaled ratio of the rotational Brownian motion to the interaction torque between cells, the scaled ratio of the adhesive bond strength to random fluctuations and the relative orientation of the adhesive bonds. These parameters together characterize the dynamics and persistence of movement in aggregates.

### (b) Sperm imaging and analysis

Captive stocks of wild-derived *Peromyscus maniculatus bairdii* and *Peromyscus polionotus subgriseus* were obtained originally from the Peromyscus Genetic Stock Center and have been maintained at the Harvard University in accordance with guidelines established by Harvard's Institutional Animal Care and Use Committee. We used adult (age more than 90 days) sexually mature *P. polionotus* (*n* = 9) and *P. maniculatus* (*n* = 9) males for cross-species comparisons.

After sacrifice, we immediately removed the left caudal epididymis of each male, made a single small incision in the tissue, submersed it in 1 ml of warmed Biggers–Whitten–Whittingham media [36], and incubated the tissue for 10 min at 37°C to release motile sperm. After the 10 min incubation, we removed the epididymal tissue, gently swirled the media and incubated for another 5 min. We collected 20 µl of media containing live sperm just below the surface of the aliquot, to reduce the number of dead cells, which sink to the bottom. We placed the aliquot on a plastic microscope slide and covered the sample with a plastic coverslip (plastic reduces adhesion of sperm to the slide compared with glass products), and recorded three 5 s videos (30 frames per second) of live sperm at 100× magnification under phase contrast conditions on an upright microscope (AxioImager.A1, Zeiss, Jena, Germany).

To examine the dynamic performance of sperm aggregates, we quantified the speed and velocity of both single cells and aggregated groups. The speed, also referred to as VCL, characterizes the rate of change of the two-dimensional projection of an aggregate's trajectory over time (figure 2). The average velocity, or VSL, is defined as the rate of change of the projected distance along the vector connecting the initial and final point in the trajectory (figure 2). We acquired VSL and VCL data from video using the computer-assisted sperm analyser plugin for NIH ImageJ [37], which tracks motile sperm cells or groups to calculate VSL and VCL. We then estimated average linearity (VSL/VCL) for each track. Specifically, for each video recorder, we first used the ‘find edges’ and ‘threshold’ functions to isolate sperm images from the background and imposed a filter to discard tracks with VSL < 5 µm s^−1^ or VCL < 25 µm s^−1^ (cut-offs imposed to avoid non-progressively motile sperm cells or groups). We then used the first 50 tracks (including both single sperm cells and sperm groups) recorded from each donor male in subsequent analyses for all but two males: in the case of one male of each species, fewer than 50 tracks met our criteria (*P. maniculatus* male, *n* = 30 tracks; *P. polionotus* male, *n* = 27 tracks). Sperm group size was then subsequently counted for each track and verified on at least five different frames per track.

We used two-factor (group size and donor male), two-tail ANOVAs to assess the effect of each factor on sperm average velocity (VSL), speed (VCL) and linearity (VSL/VCL) within each species. After identifying the sperm aggregate size that achieved the greatest average velocity (*n* = 7 cells), we then compared the average velocity of seven-celled aggregates (the null) with the average velocity of all other sizes for each species using a one-sample two-tailed *t*-test. Next, we split the *P. maniculatus* and *P. polionotus* data into two groups and used a linear regression (with donor male as a covariate) to test the significant relationship between group size and average velocity at or below the optimum (*n* ≤ 7 cells), and above the optimum (*n* > 8 cells). To identify how sperm aggregate size varies between species, we first averaged group size over each donor male, then used a two-sample two-tailed *t*-test to compare means, and an *F*-test to compare variances, of *P. maniculatus* and *P. polionotus* sperm aggregates. Finally, we used a two-way ANOVA (species and donor male) to compare difference between average linearity achieved by *P. maniculatus* and *P. polionotus* males. All statistical analyses were performed in R [38].

## 3. Results

### (a) Mathematical model

We integrated equation (2.1) numerically for a wide range of parameter values. Our model sample consists of 100 cells in a square domain of size *L* = 500 (in units of the particle minor semiaxis *b*) with periodic boundary. For all choices of parameters, aggregation always leads to a prominent increase in the average velocity, VSL (but not speed, VCL), for small aggregate size, whereas large aggregates suffer from both reduced velocity and speed (figure 3*d–i*). The origin of this behaviour can be explained by noting that sperm can associate with each other via soft adhesive bonds, modelled here as finitely extensible springs (see §2*a*). Once they are linked, they form aggregates whose structure is predominantly dictated by the geometry and the spatial distribution of the adhesive patches. The structure of the aggregates affects how the velocity of the individual sperm determines the final velocity of the aggregates. Thus, radially symmetric aggregates consisting of many sperm (e.g. figure 3*c*) are likely to be non-motile, because the velocities of the individual cells effectively cancel each other. Smaller aggregates, on the other hand, are asymmetric and maintain the typical head/tail directionality of individual sperm (e.g. figure 3*b*). More importantly, their close packed structure reduces the random fluctuations in the swimming direction of the individual cells, resulting in a persistent linearity of the trajectory and therefore a higher average velocity (VSL). The combined effect of these two competing mechanisms leads to an optimal aggregate size.

The precise value of the optimal aggregate size, as well as the sharpness of the velocity peak, depends on the detailed geometry of the head/mid-piece complex and the adhesion properties of the sperm heads. To investigate how cell geometry affects the swimming performance of an aggregate, we simulated self-propelled particles of various aspect ratios, the ratio between the length of the major and minor semiaxes of the elliptical particle. Increasing the slenderness of the particles moves the velocity optimum towards larger aggregate sizes and simultaneously reduces the slope of the speed curve (figure 3*d,g*). This is because slender elliptical particles can pack more tightly than circles in two dimensions, so that it requires a larger number of particles to reach a symmetric conformation. Increasing the adhesion range *r*_a_ (thus the stall force that a single adhesive bond can withstand) also has the effect of moving the optimum towards larger aggregates (figure 3*e*), while leaving the speed essentially unaltered (figure 3*h*). Increasing the sperm propulsion velocity *v*_0_ (figure 3*f*) affects the position of the optimum only slightly, but produces a substantial improvement in the dynamic performance of aggregate.

Finally, we note that in our two-dimensional self-propelled particles model, the aggregate size at which the speed starts to drop has a straightforward geometric interpretation related to the *kissing number* of the particles, defined as the number of particles that can touch a given central particle without overlap. If the ellipses are not excessively slender, then this number equals six (the same as for circles), thus aggregates formed by six or more ellipses tend to be highly symmetric and undergo a severe drop in speed (figure 3*g–i*). *Peromyscus* sperm cells have a flat head-shape roughly similar to a very oblate ellipsoid (figure 1*b,c*). For this type of shape, one might expect a kissing number between six and twelve, the latter being the kissing number for spheres in three dimensions.

### (b) Experiments

In both *P. maniculatus* and *P. polionotus*, motile sperm groups varied in size, ranging from 1 to 35 cells per group. We found that, overall, group size significantly influences average velocity (VSL) in both species, even after the variation between donor males is accounted for (table 1). However, there is no significant relationship between the number of sperm in a group and speed (VCL), yet, similar to the result for average velocity, we found a significant effect of donor male in both species for speed (table 1). Finally, when we measured the linearity (VSL/VCL) of all sperm groups, we found that group size significantly affects linearity, with donor male as a covariate, in both species (table 1).

**Table 1.** Two-factor two-tailed ANOVAs on sperm performance data.

measure	species	factor	*F*	d.f.	*p*-value
average velocity (VSL)	*P. maniculatus*	group size	34.3	1	9.41 × 10^−9^
		donor male	16.7	8	<1 × 10^−15^
	*P. polionotus*	group size	42.1	1	2.52 × 10^−10^
		donor male	7.6	8	1.90 × 10^−9^
speed (VCL)	*P. maniculatus*	group size	2.1	1	0.15
		donor male	5.7	8	6.82 × 10^−7^
	*P. polionotus*	group size	3.5	1	0.06
		donor male	4.9	8	8.41 × 10^−6^
linearity (VSL/VCL)	*P. maniculatus*	group size	89.4	1	<1 × 10^−15^
		donor male	17.4	8	<1 × 10^−15^
	*P. polionotus*	group size	131.4	1	<1 × 10^−15^
		donor male	16.5	8	<1 × 10^−15^

**Figure 4.**
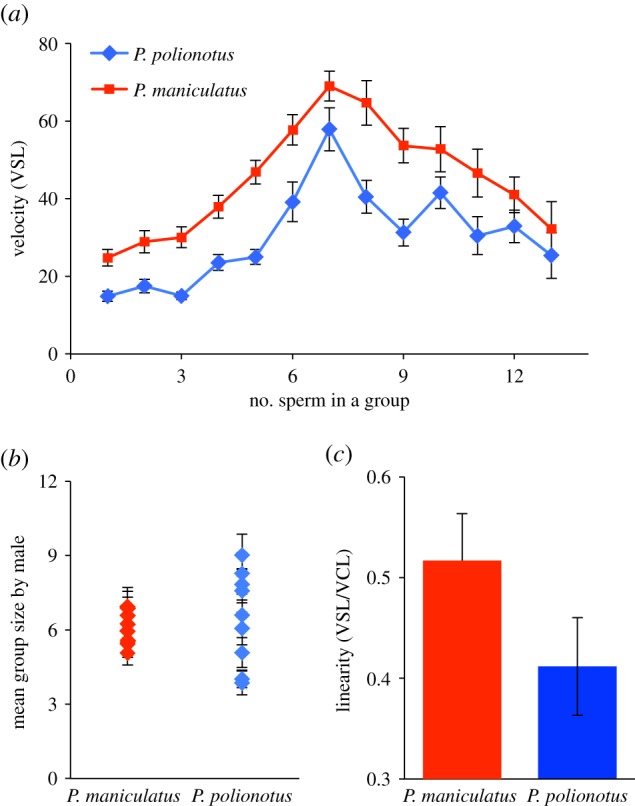
Size and performance of *Peromyscus* sperm aggregates. (*a*) Mean ± standard error (s.e.) of velocity (VSL) of sperm aggregates by group size over all donor males from each species. (*b*) Mean ± s.e. group size of aggregated sperm in each species; sperm from each donor is represented as a separate point with error bars. (*c*) Mean ± s.e. linearity (VSL/VCL) of aggregated sperm over all males from each species; note truncated *y*-axis. (Online version in colour.)

The greatest average velocity was achieved by groups of seven sperm cells (figure 4*a*) and aggregates both smaller (*n* < 6 cells) and larger (*n* > 8 cells) than this number were slower in both species (*P. maniculatus t* = 4.2, d.f. = 8, *p* = 0.003; *P. polionotus t* = 10.4, d.f. = 8, *p* = 0.0001). Moreover, we found a significant positive association between sperm aggregate size and average velocity in both species as group size increased from one to seven sperm cells (*P. maniculatus R^2^* = 0.48, *p* = 2.0 × 10^−16^; *P. polionotus R^2^* = 0.42, *p* = 2.0 × 10^−16^), yet a significant decrease as groups grew larger than seven cells (*P. maniculatus R*^2^ = 0.39, *p* = 1.63 × 10^−10^; *P. polionotus R*^2^ = 0.14, *p* = 5.20 × 10^−4^).

When we averaged sperm performance for each male in both species, we found that mean aggregate size did not differ significantly between species (mean ± s.e. = cells per group: *P. maniculatus* = 6.0 ± 0.2, range = 2–26 cells, *P. polionotus* = 6.5 ± 0.72, range = 2–31 cells, *n* = 50 groups per male, *n* = 9 males; *p* = 0.51, d.f. = 8); however, the average group size in *P. polionotus* is significantly more variable than in *P. maniculatus* (figure 4*b*; *F*-test: *p* = 0.044). Moreover, the average linearity (VSL/VCL) achieved by sperm of *P. maniculatus* males is significantly greater than the average linearity of *P. polionotus* sperm (figure 4*c*; *F* = 47.45, d.f. = 1, *p* = 1.11 × 10^−11^).

## 4. Discussion

Our combined theoretical and experimental approach allowed us to build a mathematical model based on biological observations with testable predictions. Our empirical results are consistent with the salient predictions. These include: (i) when sperm conjugate in a head-to-head formation, such as in *Peromyscus*, and when aggregate size exceeds the optimum, cells will exert opposing forces upon one another and thereby reduce the velocity of the entire group, and (ii) the optimal size is dictated largely by the geometry of the sperm heads, and therefore species with similar sperm heads, such as *P. maniculatus* and *P. polionotus* [25], will achieve the same optima. We also found that, overall, group size significantly influences average velocity (VSL) in both species, and the greatest average velocity is achieved by groups of seven sperm cells—aggregates both smaller (*n* < 6 cells) and larger (*n* > 8 cells) than this number are progressively slower in both species. Taken together, our results suggest that the shared aggregate geometry of *P. maniculatus* and *P. polionotus* sperm (probably as a result of the similarly shaped (figure 1*b,c*) and sized [25] sperm heads) results in a similar relationship between sperm group size and performance, and thus similar optima, in these species.

A third prediction of the model is that sperm aggregates achieve greater average velocity (VSL) because they move in a more linear path, rather than an increase in speed (VCL). Indeed, we found no significant relationship between the number of sperm in a group and speed in either *P. maniculatus* or *P. polionotus*. However, like velocity, we found a significant effect of donor male in both species on speed; this variation among males and between species is consistent with earlier findings of intermale differences in speed in these species (Fisher *et al*. 2014, unpublished data). By contrast, when we measured the linearity (VSL/VCL) of all sperm groups, we found a significant effect of group size on linearity with donor male as a covariate in both species. These results indicate that the benefit of sperm aggregation is, indeed, conferred via a more direct path of travel, rather than a change in speed, as predicted by our model.

Our experimental results are consistent with the predictions of the model that shared aggregate geometry of *P. maniculatus* and *P. polionotus* will yield similar relationships between sperm group size and performance in both species. In nature, however, the ideal strategy is not always the most prevalent one owing to associated costs, selection on pleiotropic traits and/or genetic drift. While *P. maniculatus* sperm have evolved under a selective regime with intense competition [21,22], evidence suggests that monogamy in *P. polionotus* [23,24] is derived [39] and, therefore, sexual selection is likely relaxed in *P. polionotus*. When we measured the average aggregate size in each male across the two species, we found that while the average group size does not differ significantly between species, the average group size in *P. polionotus* is significantly more variable than *P. maniculatus*. In other words, the mean group size does not differ between the species, which are both within one cell of the observed (empirical) and predicted (theoretical) optimum, but the distribution around the mean is significantly larger in *P. polionotus*, and thus more aggregates are further away from the optimum, compared with *P. maniculatus*. These results suggest that sexual selection, and male–male competition specifically, may be imposing stabilizing selection on aggregate size in *P. maniculatus* sperm, resulting in more groups at or near optimal size; by contrast, the monogamous mating system of *P. polionotus* represents a relaxation of male competition and is consistent with greater variation in sperm group size.

Given that *P. maniculatus* sperm are more likely to form aggregates at or near the optimal size compared with *P. polionotus*, our model also predicts an overall increased linearity in *P. maniculatus* sperm in the total sample. Indeed, *P. maniculatus* sperm move in a more direct trajectory (VSL/VCL) than *P. polionotus* sperm. The results from this study reveal that selection may, therefore, act on sperm swimming performance via aggregation behaviour.

## 5. Conclusion

Our detailed observations of sperm shape, aggregate geometry and their dynamical performance suggest an optimal sperm aggregate size that leads to a maximum linear velocity of a group. Our minimal mathematical model—that accounts for the geometry of the sperm and the mechanics of their adhesive interactions, when combined with the dynamics and fluctuations of translational and rotational movement—captures the non-monotonic dependence of aggregate velocity on the number of sperm in a group. The underlying mechanism is simple: in small groups, sperm adhesion reduces the size of rotational fluctuations by effectively cancelling them, whereas in large aggregates, this effect is dominated eventually by reducing the mean translational velocity owing to the isotropic geometry of a cluster. Thus, relatively few mechanical parameters can explain the dynamics of a seemingly complex biological process.

Our empirical data test these model predictions and show that sperm achieve greater velocity surprisingly not by increasing speed, but rather by travelling in a more direct path than solitary cells. This collective behaviour arises from direct physical interaction among cells, which determines the optimal aggregate size. The number of cells involved in an aggregate, therefore, greatly contributes to sperm performance and the reproductive success of a male, thereby offering another dimension of sperm biology on which selection can act. Moreover, by comparing sperm dynamics in two species that have evolved under disparate competitive regimes, we are able to implicate a role for sexual selection in the evolution of complex behaviour of these seemingly simple cells.

Thus, a deep understanding of sperm behaviour requires us to combine our knowledge of geometric and physical constraints with reproductive biology; indeed, these dynamics are clearly driven by a combination of morphology, kinematics and the selective environment. While selection ultimately acts on organismal fitness, our picture allows us to link this to the dynamics of movement and the adhesive interactions among sperm. Indeed, sperm cooperation and competition are a remarkable arena not only to study evolutionary processes, but also to test quantitative models for how they may play out in nature.

## Supplementary Material

Figure S1

## Figures and Tables

**Figure 5.**
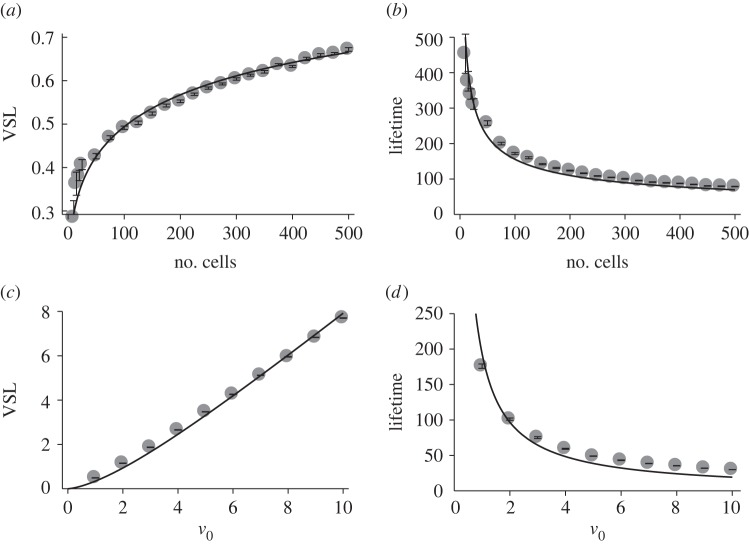
Average velocity (VSL) and lifetime (i.e. aggregation time) of a single cell. Both VSL and *v*_0_ are expressed in units of *a*/*τ*_*c*_, with *a* the length of the cell minor semiaxis and *τ*_*c*_ = 1/*D* the correlation time. The circles are obtained by numerical integration of equation (A 1), whereas the solid lines are given by equation (A 11) by setting 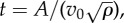 with *ρ* the particle density and *A* a fitting parameter.

**Figure 6.**
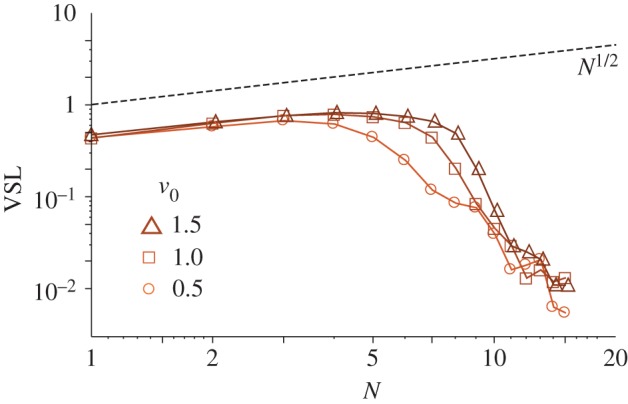
Log–log plot of the average velocity (VSL) versus aggregate size for various values of propulsion velocity *v*_0_. The numerical data are the same as in figure 3*f*, while the dashed line shows the predicted scaling law VSL ∼ *N*^1/2^ in two dimensions. (Online version in colour.)

## Appendix A

**(a) Single sperm dynamics**

To understand why aggregation improves the mobility of sperm, it is useful to consider the dynamics of a single cell resulting from equation (2.1) in the main text:

A1

where *ζ* is a zero-mean delta-correlated Gaussian random variable:  with *D* a rotational diffusion constant. Equation (A 1) describes the motion of a cell moving at constant speed *v*_0_, but whose direction of motion is affected by random rotations. Translational noise can be neglected due to the fact that the typical Peclect number of a swimming sperm of size *L* is  [40], where *D*_*t*_ is the translational diffusion constant of the sperm; thus, diffusion is negligible compared with drift. The beating of the tail that controls the orientation of the cell, on the other hand, is subjected to fluctuations owing to the noise in the activity of the motors regulating the flagellar beating. Equation (A 1) can be easily solved using the standard machinery of Brownian motion [41,42]. Integrating the *θ* equation yields

A2

from which

A3

where min(*t, t′*) represents the minimum between the times *t* and *t′*. Now, as Δ*θ* is a linear combination of random variable with zero mean and variance 〈Δ*θ*^2^(*t*)〉 = 2*Dt*, from the central limit theorem, it follows that it is Gaussianly distributed. Namely

A4

where we called *P*[Δ*θ*(*t*)] the probability density function associated with Δ*θ*(*t*). This allows us to calculate the averages of exponential and trigonometric functions

A5

from which

A6

Averaging both sides of the position equation (A 1) allows us to calculate the average velocity of a sperm cell:

A7

where ***n***(0) = (cos*θ*_0_, sin*θ*_0_) is the direction of the cell axis at *t* = 0. Equation (A 7) represents an important property of our model sperm cell: at short times, individual sperm move ballistically in the direction of their axis, but, owing to the noisy flagellar beating, their velocity exponentially loses directional correlation and in a time scale of order *τ*_*c*_ = 1/*D* the cell has completely lost track of its initial direction. Aggregation allows groups of cells to reduce the fluctuations in the direction of motion, thus increasing the correlation time *τ*_*c*_ (see later).

The mean-square displacement of a single cell can be calculated as

A8

and yields

A9

Combining this with equation (A 8) gives

A10

Thus, for  the motion is completely Brownian with  The average speed (VCL) and velocity (VSL; figure 2) of a single cell are then given as follows

A11

Because the mean-square displacement does not depend linearly on time, VSL depends in general on the time range used to calculate it and vanishes in the limit *t* → ∞, indicating that a cell has been diffusing long enough to visit its initial position more than once. For the dynamics of a single sperm cell, we can replace *t* in equation (A 9) with the lifetime *τ*_1_ of an isolated cell, defined as the time it takes for the cell to meet other cells and form and aggregate. If the sample consists of a density *ρ* of uniformly distributed cells, then this is approximately given by  Figure 5 shows the average velocity and the lifetime of isolated cells for various values of *v*_0_ and cell number obtained from numerical integration of equation (A 1).

In the experiments, the time range *t*_exp_ used to calculate VSL is fixed and is based on the frame rate of the images (30 frames per second). This can be incorporated in the definition of *D*, by introducing a dimensionless rotational diffusion number  The long-time average velocity is then characterized by two quantities:  Furthermore, the above-presented discussion is not only restricted to the case of an isolated cell, but can also be extended to aggregates. In the case of an aggregate, VCL is the average velocity of the aggregate, whereas VSL/VCL can be taken as a measure of the effective diffusion number  that accounts also for the reduction in the orientational noise resulting from aggregation.

**(b) Scaling**

The scaling behaviour of the VSL for increasing aggregate size can be predicted within a mean-field framework starting from a simple geometric argument. As explained in the previous section:  Thus, if the angular span Δ*θ* is reduced by a factor *x* < 1, then VSL is increased by a factor 1/*x*. Now, in the interior of an aggregate, Δ*θ* decreases owing to the crowded environment. To account for this effect, we use the following ansatz:

A12

where *Ω**_d_* is the solid angle in *d*-dimensions, *k_d_* the *d*-dimensional kissing number and *n* the average number of neighbours in the aggregate. Equation (A 12) implies that each new neighbour takes an equal amount of angular space until, for *n* = *k_d_*, there is no space left and the cells have reached a jammed configuration.

The number of neighbours *n* depends in general on the size *N* of the aggregate and in particular on the number of cells in the interior of the aggregate compared with those distributed along the boundary. Thus, calling *z*_i_ and *z*_b_ the average number of neighbours in the interior and on the boundary, we have

A13

where *N*_i_ and *N*_b_ represent, respectively, the number of cells in the interior and on the boundary and *N* = *N*_i_ + *N*_b_. Next, assuming *z*_i_ = *k_d_* and taking into account that *N*_b_ ∼ *N*^1/d^, we obtain

A14

with *c* a constant. Now, when the aggregate consists of a single cell, *n* = 0. This allows us to calculate the constant *c* in the form *c* = *k_d_*/(*k_d −_ z*_b_), from which  and equation (A 12) becomes

A15

Consequently

A16

For our simple two-dimensional model, this prediction is consistent with the numerical data shown in figure 6.
