# Supplementary material for: The dynamics of sperm cooperation in a competitive environment
Source: Proc Biol Sci. 2014 Sep 7;281(1790):20140296. doi: 10.1098/rspb.2014.0296 (PMC4123693; doi:10.1098/rspb.2014.0296)
Supplement: Figure S1 [file rspb20140296supp1.pdf]

Electronic Supplementary Material (ESM)

Supplemental movie captions

Movie 1: Live *P. maniculatus* sperm aggregates imaged at 400X using phase contrast light microscopy.

Movie 2: A typical solution obtained from a numerical integration of equations (1) in the main text. The cells link together to form aggregates of various sizes and geometry. Dense aggregates have radial symmetry and this reduces their motility as the cells are forced to swim against each other.

Supplemental figure

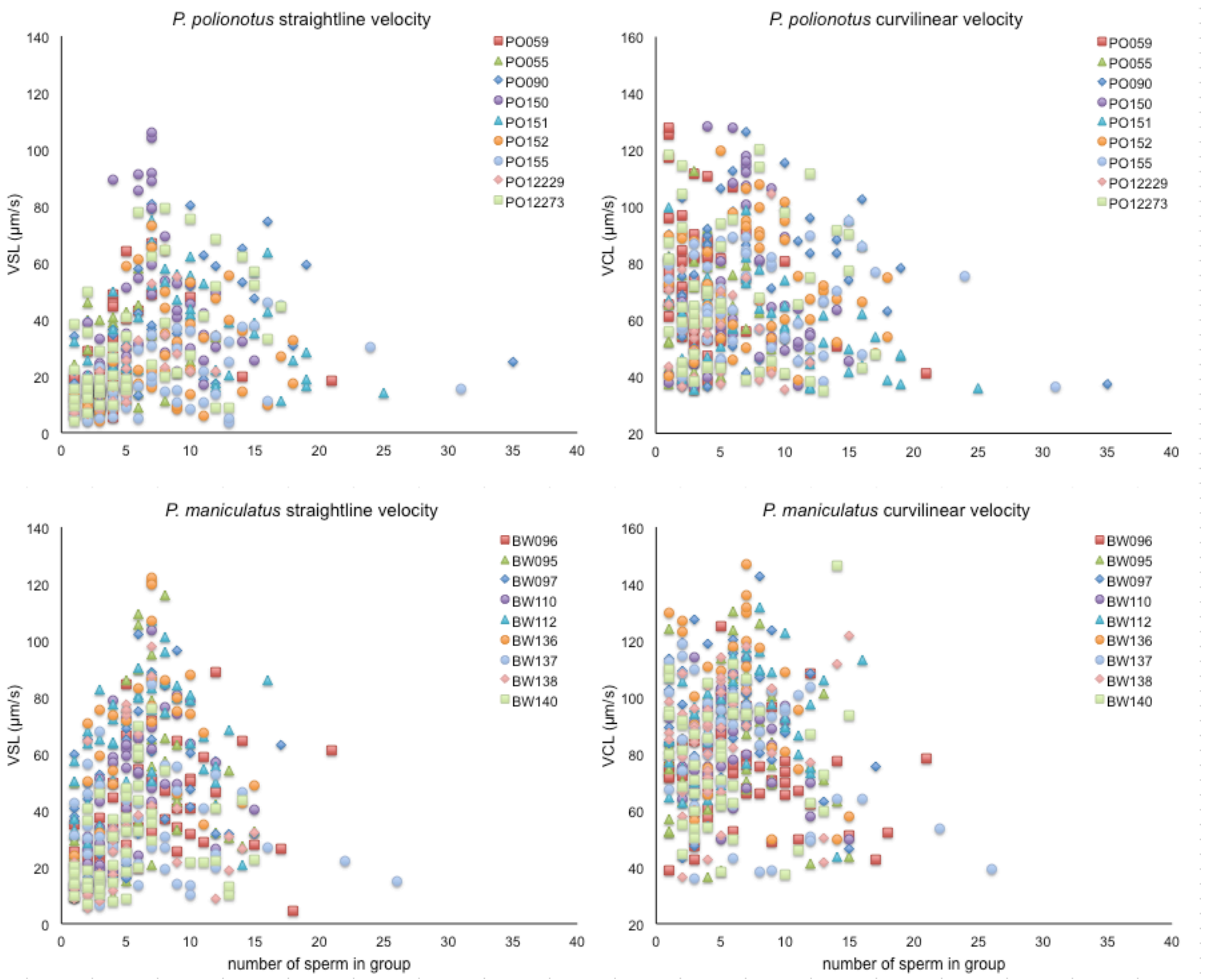

Figure S1: Straightline velocity (VSL) and curilinear velocity (VCL) of all sperm aggregates measured, labelled by donor male and indicated by the colored points.
